# Association between socioeconomic status and obesity among 12-year-old Malaysian adolescents

**DOI:** 10.1371/journal.pone.0200577

**Published:** 2018-07-25

**Authors:** Aryati Ahmad, Nurzaime Zulaily, Mohd Razif Shahril, Engku Fadzli Hasan Syed Abdullah, Amran Ahmed

**Affiliations:** 1 Faculty of Health Sciences, Gong Badak Campus, Universiti Sultan Zainal Abidin, Kuala Nerus, Terengganu, Malaysia; 2 Faculty of Informatic & Computing, Besut Campus, Universiti Sultan Zainal Abidin, Besut, Terengganu, Malaysia; 3 Institute of Engineering Mathematics, Pauh Putra Campus, Universiti Malaysia Perlis, Arau, Perlis, Malaysia; Yenepoya Medical College, Yenepoya University, INDIA

## Abstract

The epidemic of obesity in developed countries is commonly associated with poor dietary habit and sedentary lifestyle. However, other determinants, including education background and family income, may contribute towards the problem especially in developing countries. This study aimed to determine the influence of socioeconomic status (SES) on obesity among 12-year-old school adolescents in Terengganu, Malaysia. Body weight and height were measured and BMI was categorised based on WHO z-score cut-off points. Information was obtained from self-reported questionnaire on parents’ education background, family income and occupation. A total of 3,798 school adolescents aged 12 years (44% boys and 56% girls) were recruited. There was no significant difference in BMI status between boys and girls, or between rural and urban participants. There were significant differences between BMI categories and gender, household income and SES level within rural areas. In the urban areas, significant differences were found between BMI categories and gender, parents’ occupational and educational level, household income and size, and SES level. A logistic regression model found several SES factors to be predictors of obesity in this population, namely, gender, household size, father’s occupation level, household income level and SES level. Each component of SES has been significantly associated with the BMI category of school adolescents, particularly in the urban areas. This suggests the requirement of multifaceted approaches, including the role of family, society and authorities, in the effort to curtail adolescent obesity.

## Introduction

During the past three decades, the world prevalence of obesity among children and adolescents has escalated dramatically [1, 2]. In Asia, the epidemic has now become a significant public health problem, mainly in the low and middle-income countries. In Malaysia, the national prevalence of obesity has increased from 5.7% in 2011 to 11.9% in 2015 [3, 4]. Childhood and adolescence obesity imposes both short and long term negative effects on health and well-being. Children and adolescents who are obese are likely to become obese as adults [5] and are at greater risk of developing diabetes mellitus type 2, sleep apnoea, and cardiovascular, bone and joint diseases, as well as social and psychosocial problems such as stigmatisation and poor self-esteem [6].

Obesity has been associated with numerous risk factors including genetics, lifestyle and certain diseases, and medication intake. Lifestyle factors, mainly high-energy intake and reduced physical activity, have been identified as key factors leading to obesity particularly in adolescents [7]. Nonetheless, obesogenic environmental factors, including socioeconomic status (SES), have emerged as also contributing to the obesity problem, although these factors may need an extensive investigation. Through appropriate and suitable intervention programmes, obesity can be prevented and treated. A broad understanding of this epidemic and its associated factors will help to guide the proper development of population-based policies and effective intervention programmes. Although many studies have revealed the association between SES and obesity, the impact of these factors on BMI status among adolescents in Malaysia, specifically in a sub-urban state such as Terengganu, is unclear.

This study aimed to determine the influence of socioeconomic factors on the prevalence of obesity among Malaysian adolescents in Terengganu, and to provide evidence on the contribution of environmental factors towards the epidemic of obesity among the adolescents. These data can be used as a basis to develop and implement relevant intervention programmes.

## Methods

### Study design and sampling

This cross-sectional study was conducted from November, 2014 to June, 2015 and involved all 12-year old school adolescents from all government primary schools in Kuala Terengganu and Besut districts of Terengganu, a state in the East Coast Region of Peninsular Malaysia. These two districts were selected based on demographic and logistic factors as approved by the Malaysian Ministry of Education and Terengganu State Education Department. Nevertheless, after careful research prior to selection, these two districts covered both urban and rural school locations.

### Study participants

A total of 3,798 school adolescents comprising 1,667 boys (44%) and 2,131 girls (56%) participated in this study. Participants were also sub-classified based on school locations (urban vs. rural) set by the Terengganu State Education Department for analysis purposes.

### Data collection

Parental consent for students’ participation was obtained prior to the measurements. Data on height, body weight, age and gender were obtained from the 2015 National Fitness Standard (SEGAK) assessment test and uploaded into a specific database named the Health Monitoring and Surveillance System (HEMS) [8]. The SEGAK is a mandatory physical fitness test that is conducted twice a year in all government schools in Malaysia. Information on parents’ education background, family income and occupation were obtained from self-reported questionnaires. Verification of self-reported information was cross-checked with the schools’ database. The SES level was determined from these three components [9].

### Anthropometry measurements

Height and weight were measured by trained physical education (PE) teachers in each school according to the reference material and standardised protocol provided [10]. Body mass and stature were measured using calibrated analogue health scales to the nearest 0.1 kg and 0.1 cm, respectively. At the time of data collection, all participants were apparently healthy and all measurements were taken in light sports attire without shoes during mornings or early afternoons. Data on height, weight, gender, and age were used to compute the BMI-for-age Z-score using WHO AnthroPlus software [11]. The BMI was calculated by dividing the body weight in kilograms (kg) by the height in metres squared (m^2^). Teachers-measured weight and height met excellent reliability criterion (i.e. based on ICC values) suggesting that PE teachers’ measurements were reliable. The intra-class coefficient (ICC) for weight, height and BMI were 0.93, 0.98 and 0.91, respectively, which indicates substantial reliability. The BMI categories were defined using age- and sex- specific cut-off points relative to the WHO 2007 classifications [12]. The interpretation of the cut-offs classifies overweight as having a z-score > +1SD, obesity as having a z-score > +2SD and thinness as having a z-score < -2SD.

### Statistical analyses

Some SEGAK data were not available from several schools due to inappropriate data entry by the PE teachers. The total population of 12 years old school adolescents for two districts was 9,624. However, only complete returned questionnaires were considered in the analysis (*n* = 3,798). The results were examined for extreme values where reported BMIs were below -5SD and exceeded +5SD, which were the arbitrary cut-off points stipulated by NHMS [3]. Descriptive statistics were presented as means with their standard deviation, or percentage of prevalence to describe the characteristics of the participants’ mean weight, height, age and BMI. Independent sample *t*-tests were used to test differences in means of BMIs between genders and school locations (rural vs. urban). Pearson’s chi-square test was used to determine the association between BMI categories and SES levels and their components. Logistic regression reporting odds ratios (ORs) was used to determine the factors associated with obesity by comparing the non-obese (BMI z-score <+2SD) and obese groups (BMI z-score >+2SD), based on WHO 2007 growth reference [12]. Multivariable models were adjusted for gender, school locations, parents’ occupation level, parents’ education level, socioeconomic level, household income and household size. Data were analysed using IBM SPSS Statistics for Windows, Version 22.0 software (IBM Corporation, Armonk, New York, USA). A two-sided *P* value of less than 0.05 was considered as statistically significant.

### Ethics statement

This study obtained ethical approval from the Universiti Sultan Zainal Abidin Human Research Ethics Committee (UHREC) (Reference: UniSZA.N/1/628-1Jld.2 (11)). Permission to conduct the study was obtained from the Malaysian Ministry of Education and Terengganu State Education Department. Informed written consent from parents to participate in this study was obtained prior to the measurement. Consent to publish the data was obtained from the Malaysian Ministry of Education and Terengganu State Education Department.

## Results

The anthropometrical characteristics of participants are shown in Table 1. Overall, the mean BMI of boys was not significantly different to that of the girls: 18.9±4.7 kg/m^2^ and 18.8±4.3 kg/m^2^, respectively. Similarly, the BMIs of boys and girls of the urban school locations did not differ from those of their rural counterparts.

**Table 1:** Anthropometric measurements by gender.

	Boys (*n* = 1667)	Girls (*n* = 2131)	All (*n* = 3798)
**Height (cm)**	142.9 ± 8.4	145.0 ± 7.6	144.0 ± 8.0
**Weight (kg)**	39.0 ± 12.4	39.9 ± 11.0	39.5 ± 11.7
**BMI (kg/m**^**2**^**)**	18.9 ± 4.7	18.8 ± 4.3	18.8 ± 4.4
**BAZ**	0.13 ± 1.7	0.04 ± 1.5	0.08 ± 1.6
** Rural (*n* = 1846)**			
** Height (cm)**	143.1 ± 8.1	145.3 ± 7.6	144.3 ± 7.9
** Weight (kg)**	39.1 ± 12.9	39.8 ± 11.4	39.5 ± 12.1
** BMI (kg/m**^**2**^**)**	18.8 ± 4.9	18.7 ± 4.3	18.8 ± 4.6
** BAZ**	0.07 ± 0.02	-0.02 ± 1.5	0.02 ± 1.7
** Urban (*n* = 1942)**			
** Height (cm)**	142.7 ± 8.6	144.8 ± 7.7	143.9 ± 8.1
** Weight (kg)**	39.0 ± 12.1	40.0 ± 10.8	39.5 ± 11.4
** BMI (kg/m**^**2**^**)**	18.9 ± 4.6	18.9 ± 4.2	18.9 ± 4.4
** BAZ**	0.17 ± 1.7	0.08 ± 1.5	0.12 ± 1.6

BMI, Body mass index; BAZ = Body Mass Index (BMI)-for-age z-score; Data are (mean ± SD)

Most (60.7%) participants were classified as of normal weight, whereas 9.6%, 15.6% and 14.1% were classified as thin, overweight and obese, respectively (Table 1). The proportions of boys who were thin and obese were higher than girls, while the girls with normal weight outnumbered the boys. In contrast, the percentage of overweight boys and girls was similar. Nonetheless, a significant association was found between BMI categories and gender throughout all the participants (*P*<0.001, χ^2^ = 36.6) (Table 2).

**Table 2:** Percentage of BMI categories within school location.

Variables	Rural (*n* = 1846)					Urban (*n* = 1942)				
	Thin (*n* = 175)	Normal (*n* = 1130)	Overweight (*n* = 290)	Obese (*n* = 251)	*P*-value^a^ (*x*^*2*^)	Thin (*n* = 188)	Normal (*n* = 1175)	Overweight (*n* = 303)	Obese (*n* = 286)	*P*-value^b^ (*x*^*2*^)
**Gender**										
** Male**	14.0	52.3	15.9	17.9	<0.001 (34.43)	8.1	59.4	15.0	17.5	<0.001 (18.61)
** Female**	8.5	66.7	13.4	11.5		9.1	62.3	17.3	11.4	
**Household size [15]**										
** Small**	10.8	56.8	15.3	17.0	0.168 (9.11)	6.9	56.8	19.6	16.7	0.039 (13.27)
** Medium**	11.3	56.9	16.3	15.5		7.8	60.6	16.6	15.0	
** Large**	11.1	63.8	12.8	12.3		10.1	62.5	15.3	12.1	
**Mother’s occupational level [13]**										
** Fourth**	9.8	50.6	18.9	20.7	0.1 (14.68)	7.4	53.8	20.9	17.9	0.015 (20.46)
** Third**	9.5	66.7	4.8	19.0		11.1	58.3	15.3	15.3	
** Second**	12.9	56.0	13.8	17.2		9.4	57.5	18.9	14.2	
** First**	11.0	61.7	14.2	13.1		8.8	63.4	14.9	13.0	
**Father’s occupational level [13]**										
** Fourth**	7.9	51.8	20.1	20.1	0.058 (16.45)	7.5	49.6	22.4	20.5	<0.001 (46.89)
** Third**	14.5	52.7	20.0	12.7		11.4	55.1	14.2	19.3	
** Second**	11.4	63.7	13.2	11.7		8.5	66.1	15.4	9.9	
** First**	12.4	60.5	13.2	14.0		8.6	63.3	14.9	13.2	
**Mother’s education level**										
** No formal education**	10.3	72.4	3.4	13.8	0.134 (13.68)	8.8	58.8	17.6	14.7	0.011 (21.35)
** Primary education**	11.7	62.6	15.6	10.1		8.9	58.2	20.9	12.0	
** Secondary education**	11.0	60.4	14.8	13.8		8.7	63.2	15.0	13.1	
** Tertiary education**	9.8	51.5	16.7	22.0		8.5	52.5	21.5	17.5	
**Father’s education level**										
** No formal education**	17.4	63.0	8.7	10.9	0.185 (12.53)	17.4	63.0	8.7	10.9	<0.001 (34.12)
** Primary education**	8.1	67.0	15.8	9.1		8.1	67.0	15.8	9.1	
** Secondary education**	11.8	58.6	14.5	15.1		11.8	58.6	14.5	15.1	
** Tertiary education**	10.2	57.8	17.2	14.8		10.2	57.8	17.2	14.8	
**Household income level [14]**					0.011 (16.55)					<0.001 (37.34)
** Low**	11.0	62.3	15.1	11.6		8.9	63.3	16.2	11.5	
** Middle**	8.6	53.6	15.0	22.9		6.7	58.4	17.2	17.7	
** High**	10.3	53.8	15.4	20.5		8.1	47.7	22.8	21.5	
**SES level [9]**										
** Low**	12.8	62.0	13.0	12.2	0.044 (12.96)	7.4	67.1	15.0	10.5	<0.001 (40.55)
** Medium**	9.7	60.5	15.0	14.8		9.6	61.4	16.1	12.9	
** High**	10.8	51.3	17.1	20.9		8.4	50.9	19.5	21.1	

^a^BMI categories versus genders, parental occupation level, parental education level, SES level, household income level, household size within rural area (Pearson’s chi-square test)

^b^BMI categories versus genders, parental occupation level, parental education level, SES level, household income level, household size within urban area (Pearson’s chi-square test) Parental occupation classified based on MASCO 2008 (1^st^ level: Elementary jobs, 2^nd^: Administrative & operational jobs, 3^rd^ level: Technician job, 4^th^ level: Professional jobs; SES level classified based on Boey et al. (2003); Household income level (Low: <MYR 2300, Middle: MYR 2300–5599, High: >MYR5600); Household size (Small: <5 persons, Medium: 5–7 persons, Large: >7 persons)

Table 2 shows the association between BMI categories and SES in school locations (rural and urban). There was a significant association between BMI categories and gender within rural (*P*<0.001, χ^2^ = 34.4) and urban (*P*<0.001, χ^2^ = 18.6) school locations. The prevalence of thin, overweight and obese boys was higher in the rural location compared to the urban. However, the opposite trend was observed among girls, except for the prevalence of obesity. Significant associations were also found between BMI categories among urban school adolescents and mother’s occupational level (*P* = 0.015, χ^2^ = 20.5), father’s occupational level (*P*<0.001, χ^2^ = 46.9), mother’s educational level (*P* = 0.011, χ^2^ = 21.4), father’s educational level (*P*<0.001, χ^2^ = 34.1), household income level (*P*<0.001, χ^2^ = 37.3) and household size (*P* = 0.039, χ^2^ = 13.3). Among the rural school adolescents, no association was found between BMI categories and these variables, except the household income level (*P* = 0.011, χ^2^ = 16.6). The SES level was determined based on the three components of occupation, education and income level. Significant associations were found between BMI categories and SES level among both rural and urban school adolescents (*P* = 0.044, χ^2^ = 13.0 and *P*<0.001, χ^2^ = 40.6, respectively). In rural school locations, there was an association between BMI categories and three variables (gender, household size and SES level), whilst in urban areas a weak association was found with all variables (Table 3).

**Table 3:** Correlation analysis of BMI category and socioeconomic variables by school location.

Socioeconomic variables	School locations			
	Rural (*n* = 1846)		Urban (*n* = 1942)	
	*r*	*P*-value	*r*	*P*-value
**Gender**	-0.033	0.028	-0.067	0.002
**Household size**	NA	0.145	-0.086	<0.001
**Mother’s occupational level**	NA	0.089	0.091	0.03
**Father’s occupational level**	NA	0.273	0.066	0.012
**Mother’s educational level**	NA	0.171	0.084	0.028
**Father’s educational level**	NA	0.101	0.091	<0.001
**Household income level**	0.104	0.002	0.122	<0.001
**SES level**	0.106	0.026	0.089	<0.001

Data are Spearman’s rank correlations coefficients (*r*); NA: No association found between variables.

Table 4 shows that gender was moderately associated with obesity; boys were 1.6 times more likely to be obese (adjusted odds ratio (aOR) 1.66; 95% confidence interval (CI) 1.28, 2.05). An equally strong predictor was adolescents with high SES level (aOR 2.26; 95% CI 1.25, 4.06), while adolescents with a medium SES level had a minor increase in their risk of obesity (aOR 1.5; 95%CI 0.98, 2.29). The high household income group was similarly associated with obesity (aOR 1.73; 95% CI 1.04, 2.9). Nonetheless, as household size increased, the adolescents were less likely to become obese with a 36% lower risk in the medium household size group (aOR 0.64 95% CI 0.45, 0.91) and 50% less risk in the large household size group (aOR 0.5 95% CI 0.35, 0.72).

**Table 4:** Logistic regression model of factors associated with obesity among school adolescents in Terengganu, Malaysia (n = 3798).

Variables	Crude OR (95% CI)	*P*-value^a^	Adjusted OR (95% CI)	*P*-value^b^
**Gender**				
** Female**	Reference	-	-	
** Male**	1.66 (1.39, 2.0)	<0.001	1.62 (1.28, 2.05)	<0.001
**Household size [15]**				
** Small**	Reference	-	-	-
** Medium**	0.89 (0.67, 1.17)	0.379	0.64 (0.45, 0.91)	0.012
** Large**	0.69 (0.52, 0.90)	0.008	0.5 (0.35, 0.72)	<0.001
**School location**				
** Rural**	Reference	-	-	
** Urban**	0.92 (0.76, 1.10)	0.351	1.0 (0.8,1.27)	0.955
**Mother’s occupational level [13]**				
** First**	Reference	-	-	-
** Second**	1.20 (0.87, 1.63)	0.265	0.96 (0.61, 1.52)	0.861
** Third**	1.28 (0.73, 2.25)	0.386	0.78 (0.38, 1.57)	0.477
** Fourth**	1.54 (1.21, 1.95)	<0.001	0.74 (0.46, 1.19)	0.211
**Father’s occupational level [13]**				
** First**	Reference	-	-	-
** Second**	0.76 (0.59, 0.97)	0.026	0.55 (0.38, 0.8)	0.002
** Third**	1.37 (0.95, 1.99)	0.093	0.72 (0.44, 1.19)	0.197
** Fourth**	1.63 (1.25, 2.13)	<0.001	0.8 (0.51, 1.27)	0.341
**Mother’s education level**				
** No formal education**	Reference	-	-	-
** Primary education**	0.73 (0.35,1.53)	0.402	0.32 (0.08, 1.36)	0.124
** Secondary education**	0.54 (0.36, 0.81)	0.003	0.28 (0.07, 1.14)	0.075
** Tertiary education**	0.67 (0.53, 0.86)	0.002	0.28 (0.07, 1.21)	0.281
**Father’s education level**				
** No formal education**	Reference	-	-	-
** Primary education**	0.73 (0.32, 1.64)	0.443	1.68 (0.34, 8.34)	0.532
** Secondary education**	1.29 (0.62, 2.72)	0.497	2.01 (0.42, 9.65)	0.382
** Tertiary education**	1.94 (0.90, 4.16)	0.091	1.87 (0.37, 9.48)	0.449
**Household income level [14]**				
** Low**	Reference	-	-	-
** Middle**	1.81 (1.39,2.36)	<0.001	1.3 (0.91, 1.87)	0.149
** High**	2.07 (1.57, 2.72)	<0.001	1.73 (1.04, 2.9)	0.036
**SES level [9]**				
** Low**	Reference	-	-	-
** Medium**	1.24 (0.97, 1.58)	0.083	1.5 (0.98, 2.29)	0.061
** High**	2.11 (1.62, 2.74)	<0.001	2.26 (1.25, 4.06)	0.007

Data are Odds ratio (OR); 95% confidence interval (CI)

^a^Binary logistic regression

^b^ Multiple logistic regression; Dependent variable: adolescents were categorised into two groups (obese and non-obese) using WHO 2007. Parental occupation classified based on MASCO 2008 (1^st^ level: Elementary jobs, 2^nd^: Administrative & operational jobs, 3^rd^ level: Technician job, 4^th^ level: Professional jobs; SES level classified based on Boey et al. (2003); Household income level (Low: <MYR 2300, Middle: MYR 2300–5599, High: >MYR5600); Household size (Small: <5 persons, Medium: 5–7 persons, Large: >7 persons)

## Discussion

Obesity is a well-known public health problem among the world’s population, including adolescents. Many factors have been associated with the epidemic of obesity; particularly the imbalance between energy intake and energy expenditure. However, other factors such as SES may play a substantial role in the rise of obesity in adolescents. To our knowledge, this is the first study to investigate the association between SES and BMI categories among school adolescents in Terengganu, Malaysia and to compare the rural and urban school locations. Surprisingly, the present study showed no difference in the mean BMI between boys and girls, either as a whole or in rural and urban school locations. This is in contrast with the SEANUTS study, in which a significant difference in the mean BMI was found between boys and girls [16]. Similarly, this trend was also found in another study conducted in Selangor [17]. The disparity between the present findings and the previous studies might be explained by the difference in the study population. While the SEANUTS study was based on adolescents aged 7 to 12 years old, and the study in Selangor was among children aged 9 to 10 years, this present study was only conducted among 12-year old adolescents. In addition, no difference was found in the mean BMI between urban and rural adolescents. This is also contrary to the findings of previous national and state level studies [3, 16, 17].

However, regarding prevalence, this study found a significant association between BMI categories and gender. Consistent with the NHMS 2011 study, the prevalence of obesity was higher in boys than girls [3, 18]. Zalilah *et al*. also reported similar trends in obese adolescents aged 10 to 15 years old [19], but Turkish adolescents showed no association between gender and prevalence of overweight and obesity [20]. The gender difference between boys and girls may be explained by physiological changes and difference in lifestyle at this age [21]. In general, girls tend to have higher BMI as a result from rapid growth and physical changes associated with early puberty and sexual maturation. Additionally, they may engage in less physical activity and sports compared to boys. In spite of that, the prevalence of obesity was more pronounced among boys in this study. While girls were generally more cautious and restrictive about their diet [22], boys on the other hand, may consume larger meals and energy [16]. A study conducted among central and northern Malaysian adolescents found that boys consumed 10.1% higher energy compared to female adolescents [23]. Similar finding was also reported in a previous longitudinal study, the Young Heart Project [24], in which boys aged 12 to 15 years had a significantly higher intake of energy compared to their counterparts.

High SES has also been associated with an increased prevalence of obesity among children in other developing countries [25]. In agreement with the literature, the present study showed that SES may have an association with the BMI status of adolescents. The prevalence of obesity was slightly higher among rural adolescents of parents with fourth level occupations, mothers with tertiary education backgrounds and fathers with secondary education backgrounds, although these did not achieve statistical significance. In contrast to the rural adolescents, there were significant associations between BMI categories and each of the SES components among the urban adolescents. Likewise, the prevalence of obesity was also higher among the urban adolescents with parents with fourth levels of occupation. Conflicting with the study by Samani-Radia & McCarthy (2011) [26], the prevalence of obesity in this study was also highest among the adolescents of mothers with tertiary education and families with a high household income level and small household size. Nevertheless, consistent with the Turkish study [20], the highest prevalence of thinness was found in the groups with lowest household income and largest household size. As reported previously [3, 27], the prevalence of obesity increased as household income level increased. This suggests the contribution of family income to influence the eating behaviour and dietary intake pattern among family members. In addition, when measuring the SES level based on the three components described above, the highest prevalence of obesity was reported in high income groups in both urban and rural school locations, respectively. This finding contradicted that of a study of 12 to 15 years old in North Gaza which found that boys from both low and high SES had the highest risk of overweight [28].

In the present study, several SES factors were found to be predictors of obesity in adolescents. A direct link was found with household income, whilst household size showed an inverse relationship with the BMI status among adolescents which accords with the findings from previous studies [17, 26]. Higher household income and smaller household size have been reported to be associated with higher purchasing power and food affordability [29]. Furthermore, a higher parental education level, in most cases, reflects a higher family SES. Contrary to the findings in another study of Malaysian adolescents, the higher prevalence of obesity among higher SES adolescents may be explained by the higher percentage of working mothers in the present study [30]. Behavioural aspects and upbringing are shaped at home; having a working mother may affect the risk of obesity [31] because, generally, mothers are more responsible for the dietary intake and activity of their children than fathers; working mothers, especially blue collar workers [32], may have less time to spend in taking care of their children. As a result, they may have less control over their children’s food intake, eating habits and physical activity. Longer working hours of mothers has been shown to be associated with an increase in the BMI of their children [33]. However, Hofferth and Curtin (2005) suggested that working mothers’ contributions to the household income may also change their children’s lifestyle by providing a greater purchasing ability for healthy and nutritious foods and participation in structured sports [34].

This study adds to the evidence on associations between SES and BMI categories, particularly in Terengganu, Malaysia. Very limited data have been published from this state regarding adolescence obesity and its associated factors. This study has demonstrated the role of gender, family factor (father and family size) and the impact of related socioeconomic factors (father’s second occupational level and household income). Family members, especially the parents, have important equal roles in the provision of meals as well as shaping their children’s eating and physical activity habits [35]. The researchers have confirmed that fathers with better jobs and salary fail to provide adequate monitoring of food intake among their children [35]. Increases in the level of career, especially in father, have also increased the demand for away-from-home outside food[36]. This evidence can be used as a basis to develop appropriate public health policies and intervention programmes to specific target populations in order to combat obesity. Any intervention efforts to curtail adolescence obesity should directly involve the parents at the earliest stages of childhood development to ensure healthy practices, at home or elsewhere. One of the limitations of this study is that, unlike the previous national reports [3, 4], this present study does not provide any evidence on the potential role of ethnicity in obesity due to the lack of participants from other races, such as Chinese and Indian, in Terengganu. Nonetheless, it is highly recommended that similar studies be conducted in other states of Malaysia as well as in other Asian countries to determine any ethnic influences in this problem. In addition, other risk factors, such as dietary intake and physical activity, should also be measured and interpreted to determine the major causes of this epidemic among adolescents.

## Conclusions

This study highlights the influence of each component of SES, primarily education, family income and occupational status of the parents, on BMI categories of school adolescents particularly in urban areas. There is a critical need for multifaceted and community-wide approaches including the role of family, society and authorities in the effort to prevent and control adolescent obesity. Parents act as the important forces to change and inform their children's behaviours. Nonetheless, further prospective studies should be conducted examining other risk factors to determine the real causes of obesity among adolescents.
